# Investigating modifiable risk factors associated with ideal cardiovascular health among cancer survivors: a scoping review

**DOI:** 10.1186/s40959-025-00329-2

**Published:** 2025-03-31

**Authors:** Wing Lam Tock, Yujia Tang, Lise Gauvin

**Affiliations:** 1 Centre de Recherche du Centre Hospitalier de l’Université de Montréal (CRCHUM), Montréal, Québec Canada; 2 École de Santé Publique, Département de Médecine Sociale et Préventive, Université de Montréal, Montréal, Québec Canada; 3 Ingram School of Nursing, Faculty of Medicine and Health Sciences, McGill University, Québec, Montréal Canada

**Keywords:** Ideal cardiovascular health, Cancer survivors, Cardiovascular outcomes, Cardio-oncology, Lifestyle behavior, Cardiovascular risk factors

## Abstract

**Background:**

Cancer survivors are at higher risk of developing cardiovascular diseases and face worse morbidity and mortality outcomes than the general population. The American Heart Association (AHA) introduced the Life’s Essential 8 framework, encompassing eight modifiable risk factors and lifestyle behaviors for maintaining ideal cardiovascular health (CVH). Although this framework is well-established for predicting CVH in the general population, studies on its association with cardiovascular outcomes among cancer survivors remain scattered across the literature.

**Objective:**

This review maps existing literature surrounding modifiable risk factors, lifestyle behaviors, CVH, and cardiovascular outcomes among cancer survivors to take stock of what is known, identify methodological strengths and weaknesses, and propose promising research directions.

**Methods:**

A scoping review was conducted to identify studies examining different dimensions of ideal CVH in adult cancer survivors. Measurement methods of ideal CVH metrics, and determinants associated with CVH were examined.

**Results:**

Twenty-two articles met eligibility criteria. Of which, 82% (*n* = 18) were published in or after 2020. Fourteen studies (about 64%) followed the AHA’s framework to conceptualize ideal CVH. Higher scores on ideal CVH are linked to better cardiovascular outcomes among cancer survivors with associations noted for social inequalities and neighborhood environmental factors, underscoring the complexity of CVH determinants in this population.

**Conclusions:**

Research on ideal CVH among cancer survivors appears to have accelerated in recent years, yet many gaps remain to orient clinical and public health practice. Promising research directions include expanding investigations into pre-diagnosis CVH, addressing disparities in CVH across diverse populations, and conducting longitudinal studies to clarify causal pathways between lifestyle behaviors, cancer treatments, and cardiovascular outcomes.

**Supplementary Information:**

The online version contains supplementary material available at 10.1186/s40959-025-00329-2.

## Introduction

### Background

Cardiovascular diseases (CVDs) represent a leading cause of morbidity and mortality worldwide, posing a significant challenge to public health. Among cancer survivors, this burden is even more pronounced. Cancer survivors are at an elevated risk of developing CVDs and face an increased risk of premature CVD mortality than the general population [1, 2]. These risks are amplified by both the direct effects of cancer therapies, and the interplay of various modifiable and non-modifiable risk factors, such as sedentary lifestyles and pre-existing metabolic conditions [3]. Despite advancements in cancer treatment that have significantly improved survival rates, the long-term health of cancer survivors remains compromised by increased risk of cardiovascular complications [4]. The dual burden of managing cancer and the long-term effects of treatment, alongside the risk of developing CVDs, necessitates a tailored approach to cardiovascular health (CVH) assessment and management.

The American Heart Association (AHA) introduced Life’s Simple 7 (LS7) in 2010 as a framework that identifies seven modifiable risk factors for measuring and maintaining ideal CVH [5]. In 2022, the AHA updated the ideal CVH framework to Life’s Essential 8 (LE8), addressing limitations of LS7 by adding sleep health as an eighth metric, thus providing a more comprehensive approach to CVH assessment [6]. The eight LE8 modifiable risk factors include: four lifestyle behavioral factors including physical activity, diet quality, nicotine exposure, and sleep health; and four health (biological) factors including body mass index (BMI), blood lipids, blood glucose, and blood pressure [6]. The AHA developed this framework, which also includes standardized metrics for assessing LE8 (see Table 1 and supplementary material in [6]) with the goal of reducing the incidence of CVDs across diverse populations. Although patterns of LS7 and LE8 have been studied and validated in the general population, patterns among cancer survivors remain underexplored. Further, systematic reviews show that although maintaining ideal CVH across the lifespan is fundamental to preventing CVD, the prevalence of ideal CVH in adults is low [7, 8]. Nevertheless, fewer studies are conducted among cancer survivors, a population at elevated risk of late-onset CVDs.

Addressing CVH among cancer survivors is inherently complex due to the interplay of various individual and social factors that shape their health outcomes. Health inequalities significantly complicate efforts to promote CVH among cancer survivors, with socially and economically disadvantaged groups experiencing higher risks of adverse cardiovascular outcomes [9]. Cancer survivors from disadvantaged socioeconomic backgrounds often face barriers to accessing healthcare and preventive services, which exacerbate their vulnerability to both cancer-related and cardiovascular complications [10]. Moreover, marginalized populations including ethnic minorities, indigenous populations, sex and gender minorities, rural populations, and immigrants remain underrepresented in existing cardio-oncology care research [11]. These disparities highlight the urgent need for prevention strategies that address the specific cardiovascular risks of diverse survivor populations, thereby promoting more equitable health outcomes. To address the disparities in cardio-oncology care research, a comprehensive review is required to map existing evidence on CVH in cancer survivors and identify areas where further research is needed.

### Rationale for a scoping review

We opted to conduct a scoping review because this methodology is well-suited for synthesizing evidence on complex and heterogeneous topics, particularly when research is emerging, scattered across different areas of practice and disciplines, and diverse in terms of study designs, populations, and outcomes [12]. As a result, a scoping review is the most appropriate methodology to provide a comprehensive mapping of the literature, systematically identifying gaps that may not be captured through more narrowly focused systematic reviews [13]. 

### Objective

This scoping review maps existing literature surrounding modifiable risk factors, lifestyle behaviors, CVH, and cardiovascular outcomes in cancer survivors to take stock of what is known, identify methodological strengths and weaknesses, and propose promising research directions.

## Methods

We followed the Joanna Briggs Institute’s scoping review methodological framework [12] and Arksey and O’Malley’s methodological approach to promote the accuracy and reproducibility of this review [14]. Our scoping review was also conducted in accordance with the Preferred Reporting Items for Systematic Reviews and Meta-Analyses Extension for Scoping Reviews (PRISMA-ScR) checklist [15]. Critical appraisal and risk of bias assessment of studies are not mandatory in scoping reviews and so these steps were not conducted [12]. The study protocol was registered through the Open Science Framework platform (10.17605/OSF.IO/ZW36U) [16].

To ensure clarity and consistency in the review, key terms and concepts were operationally defined (Table 1). According to the National Cancer Institute, cancer survivors are defined as individuals with cancer from the time of diagnosis until the end of life [17]. Cardiovascular outcomes encompass a broad spectrum of conditions, including but not limited to coronary artery disease, heart failure, arrhythmias, stroke, and cardiovascular-related mortality [18]. Finally, CVH was operationalized in accordance with the AHA’s LE8 or LS7 framework, and other established methodologies described in the literature, providing a standardized approach to assessing modifiable risk factors and overall cardiovascular risk.

**Table 1:** Operational definitions of key terms and concepts

Key term/ Concepts	Definitions
Cancer survivors	Defined as individuals with cancer from the time of diagnosis until the end of life [17]
Cardiovascular outcomes	Encompass a broad range of cardiovascular conditions (i.e., CVDs), including but not limited to coronary artery disease, heart failure, arrhythmias, stroke, and cardiovascular-related mortality [18]
Ideal cardiovascular health	Operationalized using the AHA’s LE8 or LS7, or other frameworks. Each of the CVH metrics in the studies included in the review will be carefully examined and documented.

### Inclusion and exclusion criteria

We used the population, concept, and context (PCC) framework [19] to undertake the scoping review; the PCC uses an evidence-synthesis review design that permits broad inclusion criteria of all studies surrounding ideal CVH in cancer survivorship literature (Table 2). Adult cancer survivors aged 18 years and older were the primary targeted study population in our review, while the exclusion criteria specified cancer survivors under 18 years of age (i.e., children or adolescents). In terms of concept, we included studies that either adhered to the AHA’s framework (either LE8 or LS7) to examine pre- or post-diagnosis CVH and their associated outcomes or factors. Additionally, studies not adopting the AHA’s framework but investigating at least two LE8 lifestyle behavior metrics and two health metrics were also included in the review. Excluded were studies solely centered on single health factors related to CVH, such as body mass index or biomarkers, and those focused on the effects of cancer treatment agents and dosages on cardiovascular outcomes. Finally, we included sources with any study types, such as reviews, experimental and observational studies. Studies across all healthcare settings and populations, irrespective of gender, race, nationality, and cancer types were considered. Excluded publications include commentaries, abstracts, conference proceedings, and documents without full-text availability.

**Table 2:** The population, concept, and context mnemonic, inclusion and exclusion criteria

Term	Inclusion criteria	Exclusion criteria
Population	• Adult cancer survivors (age ≥ 18 years)	• Cancer survivors under 18 years (children or adolescent populations)
Concept	• Studies examined either pre- or post-diagnosis CVH (as estimated by a composite CVH measure such as the Life’s Essential 8 or Life’s Simple 7) and its associated outcomes/ factors • If the study did not follow AHA’s framework, we include studies that examined at least 2 LE8 lifestyle behavior metrics, and 2 health metrics	• Studies focused only on health factors associated with CVH (e.g., body mass index, blood lipids, blood glucose, and blood pressure, metabolic markers, biomarkers of cardiometabolic complication) • Studies focused on cancer treatment agents and dosage’ effects on cardiovascular outcomes
Context	• Any type of sources (experimental and quasi-experimental studies, observational studies, cross-sectional studies, qualitative studies, reviews, expert opinion papers, reference lists of key studies) • Studies conducted in all types of healthcare contexts • Studies conducted among cancer survivors irrespective of gender, race, nationality, and type of cancer	• Commentaries, abstracts, conference proceedings, and sources without available full texts

### Search strategy

Literature search strategies were conducted using medical subject headings, such as MeSH and text key words related to cancer survivors and CVH. First, an initial limited search of Ovid-Medline (PubMed) was undertaken to identify articles on the topic. The text words contained in the titles and abstracts of relevant articles, and the index terms used to describe the articles were used to develop a full search strategy for the Cochrane Library (Supplemental Table 1). The search strategy, including all identified keywords and index terms, was adapted for each included database (available on the OSF depository). The reference list of all included sources of evidence was screened to identify those appropriate for inclusion (e.g., references of studies included in the systematic review, or references of systematic reviews on the same or similar topic). We searched the following databases from the inception of each database to the date of the search: (August 22, 2024: PubMed, Ovid Embase, Ebsco CINAHL, and the Cochrane Library). No date limit was used in the searches and studies were limited to the English language.

### Source of evidence selection

**Fig. 1:**
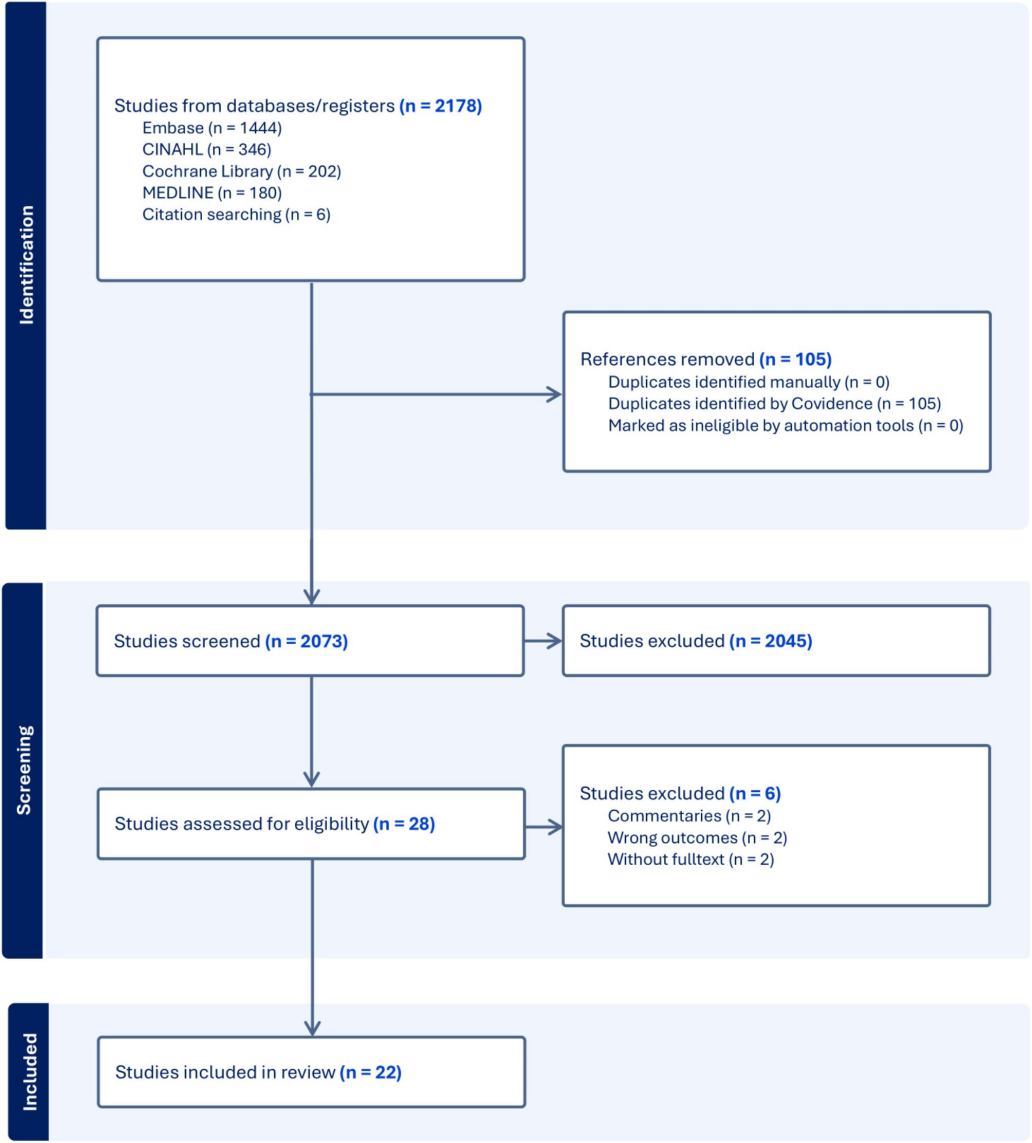
Preferred Reporting Items for Systematic Reviews and Meta-Analyses Extension for Scoping Reviews (PRISMA-ScR) flow diagram

Following the search, all identified articles were collated and uploaded into Covidence [20] and duplicates removed. Two independent reviewers (W.L.T. and Y.T.) screened the titles of the records to determine which studies were to be assessed further through Covidence. The full text of selected records was assessed in detail against the inclusion criteria by the two reviewers. Reasons for exclusion of sources of evidence at full text that do not meet the inclusion criteria were documented. We resolved discrepancies through consensus or recourse to a third review author (L.G.). Finally, in accordance with PRISMA-ScR guidance, we presented a PRISMA-ScR flow diagram showing the study selection process (Fig. 1).

### Data extraction

Data were extracted by one reviewer (W.L.T.) from included papers using a data extraction tool developed by the reviewers (Supplemental Table 2). The data extracted included specific details about the participants, concept, context, study methods, and key findings relevant to the review questions. The data extraction tool was first tested (by W.L.T. and Y.T.) on five evidence sources to ensure consistency, reliability, and appropriateness [19].

### Data synthesis

We evaluated the extracted data summarized in narrative and tabular format where appropriate. To map the available evidence, we adopted a descriptive synthesis approach [21]. This approach was chosen to adopt a broad perspective on the available evidence. The data extracted included in this review was synthesized to address the following questions:

► What are the characteristics of the included articles?

► How was CVH conceptualized and measured in the included studies?

► How were CVH metrics measured in the included studies?

► What were the findings of the included studies?

## Results

Figure 1 shows the PRISMA flow diagram summarizing the results of our systematic literature search. Results from databases and manual reference list search included 2178 records. After removing 105 duplicates, we screened 2073 records, of which 2045 were excluded by inspection of title and abstract, and 28 were assessed as full text. Of these, six were excluded for various reasons (Fig. 1). We included 22 articles in the scoping review.

### What are the characteristics of the included articles?

Table 3 displays the full descriptions of article characteristics. Of the 22 included articles, 18 (82%) were published in or after 2022. Nine (41%) were cross-sectional studies, three (14%) were retrospective cohort studies, and six (27%) were prospective cohort studies. The review also included four studies related to the development of a CVH assessment tool named Automated Heart Health Assessment (AH-HA) tool, comprising a protocol [22], a pilot usability study [23], and two cross-sectional studies [24, 25]. A majority of the included studies were conducted in the United States (82%, *n* = 18), followed by Australia, Japan, the United Kingdom, and China (*n* = 1 each). Although most studies did not clearly define cancer survivorship, commonly used definitions include at least six months following potentially curative treatment or having a confirmed diagnosis of primary cancer. Others identified survivors based on time since diagnosis or the absence of active disease.

**Table 3:** Characteristics of articles included in the scoping review (*N* = 22)

First author’s last name, year, journal	Country	Study design	Cancer survivorship definition	Cancer survivors’ characteristics: sample size (*N*), diagnosis, sex, age, ethnicity, education, income	Data source/Recruitment methods	CVH definition/ conceptualization/ measurement	Method and timing of CVH assessment
Enright, 2010, Cancer Causes Control [37]	United States	Cross-sectional analysis	Participants reported a diagnosis of cancer more than one year prior to the survey	*N* = 1,227 Mixed cancer types; 65.5% female; Median age: 70 (57–79 years); 85.3% non-Hispanic white; 49.0% attained more than high school education; 49.9% household income <$35,000	Data from the National Health and Nutrition Examination Survey (NHANES), 1999–2006, cancer survivorship status was self-reported	Five key modifiable cardiac risk factors: blood pressure, weight, cholesterol, smoking status, exercise	Self-reported, lab tests CVH assessed post-diagnosis and treatment
Hawkes, 2011, European Journal of Cancer [38]	Australia	Prospective cohort	Participants had histologically confirmed diagnosis of a first, primary colorectal cancer	*N* = 1,966 (baseline) Colorectal cancer; 59.8% male; Age: 20–80 years; 39.4% attained 8–11 years of education; Ethnicity & income not reported	Participant identified from the Queensland Cancer Registry between 1 January 2003 and 31 December 2004	Five lifestyle factors: physical activity, television viewing; smoking, alcohol consumption, body mass index (BMI)	Self-reported Four interviews timepoints (5-, 12-, 24- and 36-months post-diagnosis)
Weaver, 2013, Journal of Cancer Survivorship [42]	United States	Cross-sectional analysis	Participants were identified as survivors if they had a primary diagnosis of breast, prostate, colorectal, or gynecologic (endometrial or ovarian)	*N* = 1,582 Mixed cancer types; 50.4% female; Age: 46.8% 65–79 years 50.4% non-Hispanic white; 36.2% some college or technical school; Income not reported	Participants were recruited from a random sample of 6,391 survivors from the Los Angeles County Cancer Surveillance Program (LA CSP) and the Cancer Prevention Institute of California (CPIC) Surveillance, Epidemiology, and End Results (SEER) cancer registries, cancer survivorship status was self-reported	Six CVD risk factors: smoking, BMI, physical inactivity, hypercholesterolemia, hypertension, diabetes	Self-reported using paper-based questionnaires CVH assessed post-diagnosis and treatment
Pelser, 2014, Cancer [39]	United States	Retrospective cohort	Not defined	*N* = 4213 colon cancer *N* = 1514 rectal cancer Over 50% male; Age: 50–71 years; < 50% college education; Race/ethnicity and income not reported	Participants from the National Institutes of Health-American Association of Retired Persons (NIH-AARP) Diet and Healthy Study cohort Cancer cases were ascertained by linkage to state cancer registries in the study area, and areas where participants tended to move during follow-up	Five lifestyle factors: healthy diet, BMI, physical activity, alcohol consumption, smoking	Self-reported using mailed, paper-based questionnaires Baseline questionnaire was completed prior to cancer diagnosis
Song, 2020, Cancer Medicine [41]	United States	Cross-sectional analysis	Not defined	*N* = 1,026 cancer survivor-spouse dyad Survivors: Mixed cancer types; 50.25% male; Mean age (SD) = 62.2(0.5); 89.28% White; 28.55% GED or high school degree; 52.14% categorized as high income	Data from the Medical Expenditure Panel Survey (MEPS) between 2010 and 2015 Cancer survivorship status was self-reported, Spouses were linked to the survivors by a spousal identifier if survivors reported being married	Three health behaviors: smoking, physical activity, diet intake Three major CVD risk factors: hypertension, high cholesterol, diabetes	Self-reported, in-person interviews CVH assessed post-diagnosis and treatment
Foraker, 2021, Contemporary Clinical Trials Communications [22]	United States	Study protocol of the Automated Heart Health Assessment (AH-HA) tool	Six months post-potentially curative cancer treatment	Target *N* = 600 Mixed cancer types	Will be recruited from 12 National Cancer Institute (NCI) Community Oncology Research Program practices	AHA’s Life’s Simple 7 framework	Self-reported, medical record CVH assessed post-diagnosis and treatment
Weaver, 2021, Journal of Medical Internet Research - Cancer [23]	United States	Pilot usability and acceptability study of the AH-AH tool	At least 3 months after potentially curative cancer treatment, excluding maintenance hormonal therapy	*N* = 49 Breast cancer; female; Age: 43% ≥ 65 years; 84% White; 47% college graduate Income not reported	Participants were identified through clinic appointment schedules and contacted by a research member (by telephone or in-person)	AHA’s Life’s Simple 7 framework	Self-reported, medical record CVH assessed post-diagnosis and treatment
Coughlin, 2022, The American Journal of Cardiology [36]	United States	Cross-sectional analysis	Not defined	*N* = 42,230 Mixed cancer types; 60.24% female; Age: 53.95% > 65 years; 75.46% White; 32.03% attended some college; 37.28% household income $50,000+	Data from the 2019 Behavioral Risk Factor Surveillance System, cancer survivorship status was self-reported	Five risky health behaviors associated with CVH: current smoking status, heavy alcohol consumption, no consumption of fruits/vegetables per day, physical inactivity, and obesity	Self-reported, telephone-based survey CVH assessed post-diagnosis
Kaneko, 2022, European Journal of Preventive Cardiology [26]	Japan	Retrospective cohort	Not defined	*N* = 53,974 Mixed cancer types; 37.8% male; Median age: 54 years; Japanese; Education & income not reported	Data from Japan Medical Data Center (JMDC) Claims Database (Tokyo, Japan) between January 2005 and April 202. Participants were identified using the International Classification of Diseases (ICD)-10 codes	AHA’s Life’s Simple 7 framework (modified), Life’s Essential 8 was also calculated and used for supplementary analysis	Health check-up records & self-reported questionnaires CVH assessed > 1 year after the diagnosis of cancer, including the initial health check-up, and 1 year after the health check-up (up to 5 years)
Chan, 2023, European Journal of Preventive Cardiology [27]	United States	Cross-sectional analysis (research letter)	Not defined	*N* = 13,485 Mixed cancer types; 57% female; Age: >18 years; 88% White; 71.2% family income ≥ 200% of poverty threshold; Education & income not reported	Data from National Health Interview Survey (NHIS), cancer survivorship status was self-reported	AHA’s Life’s Essential 8 framework (without dietary data)	Self-reported CVH assessment timing not specified
Connor, 2023, Frontiers in Public Health [35]	United States	Cross-sectional analysis	Not defined	*N* = 100 Black/ African American breast cancer survivors; Mean age (SD, range): 58.6 (10.1, 32–78); 36% 4-year college degree; 26% annual household income $100,000 or more	Participants recruited from social media platform (click-oriented ads) via Meta and Instagram began from January to August 2022, breast cancer survivorship status was self-reported	Five behavioral/lifestyle factors: current BMI, BMI at breast cancer diagnosis, and BMI at age 25 years, smoking status, alcohol consumption, diet/nutrition, physical activity	Self-reported, online survey CVH assessment timing not specified
DeMari, 2023, Gynecologic Oncology [24]	United States	Cross-sectional analysis (part of the AH-HA study)	Six months post-potentially curative treatment for currently without evidence of disease	*N* = 55 Endometrial cancer; Female; Mean age = 62; (IQR 53, 70); 87% non-Hispanic White; 43.6% College graduate or post-graduate; 96.4% no financial hardship	Data from an ongoing trial of the AH-HA tool conducted through the NCI Community Oncology Research Program	AHA’s Life’s Simple 7 framework	Self-reported (diet & physical activity), and collected during health check-up appointment after diagnosis
Peng, 2023, Nutrients [40]	United Kingdom	Prospective cohort	Not defined	*N* = 13,348 Breast cancer; Mean age (SD) = 58.80 (7.1); 95.7% White European; 56.7% education > 15 years; 45.3% income < 30,999	Data from the baseline survey of UK Biobank (conducted from 2006 to 2010 across the United Kingdom, cancer survivorship status was self-reported, and identified using operation information, and ICD codes	“Healthy lifestyle score” with five modifiable lifestyle factors: BMI, smoking, alcohol drinking, dietary habits, and physical activity	Self-reported, standardized electronic questionnaire CVH assessment timing not specified
Zhang, 2023, European Journal of Preventive Cardiology [34]	China	Prospective cohort (research letter)	Not defined	*N* = 4,424 Mixed cancer types; 78% male; Age: 58% ≥60 years; Chinese; Education & income not reported	Data from the Kailuan cohort. All participants underwent questionnaire assessments, physical examinations, and laboratory tests at 11 hospitals affiliated with the Kailuan Group Following health checkup surveys were conducted every two years. Participants were newly diagnosed with cancer between 2006 and 2020	AHA’s Life’s Essential 8 framework (modified)	Standard questionnaires through face-to-face interview by trained professionals Anthropometric measurements and blood tests were conducted according to standard protocols CVH assessment timing not specified
Fan, 2024, Preventive Medicine [28]	United States	Retrospective cohort	Not defined	*N* = 1,818 Mixed cancer types; 59.1% female; Age: 6.7% 40–64 years; 67.88% non-Hispanic White; 72% high school or more; 54.8% poverty income ratio % ≥ 3.5	Data from the NHANES, 2005–2018, cancer survivorship status was self-reported	AHA’s Life’s Essential 8 framework	Self-reported, lab tests CVH assessment timing not specified
Lefferts, 2024, Journal of the American Heart Association [29]	United States	Cross-sectional analysis	Not defined	*N* = 172 Mixed cancer types; 58% female; Age = 74 ± 6 years; 95.9% non-Hispanic White; 46% Graduate/ professional degree; 31.9% income ≥$100,000	Data from the Physical Activity and Aging Study, an ongoing prospective cohort study of men and women at least 65 years of age, participants with a history of cancer were included in the present study	AHA’s Life’s Essential 8 framework	Self-reported, lab tests CVH assessment timing not specified
Lopez-Bueno, 2024, Current Problems in Cardiology [30]	United States	Prospective cohort	Not defined	*N* = 1,701 Mixed cancer types; 56% female; Age: 62.0 ± 15.0; 65.4% non-Hispanic White; 30.3% College graduate or above; Family income to poverty ratio: 3.03	Data from six consecutive waves of the NHANES (2007–2018), cancer survivorship status was self-reported	AHA’s Life’s Essential 8 framework	Self-reported, lab tests CVH assessment timing not specified
Qiu, 2024 BioMed Central (BMC) - Public Health [31]	United States	Cross-sectional analysis	Not defined	*N* = 2,542 Mixed cancer types; 57.34% female; Age: 62.58 ± 0.38 87.78% non-Hispanic White; 95.99% high school or above; Poverty-income ratio: 3.36 ± 0.05	Data from NHANES (2005–2018), cancer survivorship status was self-reported	AHA’s Life’s Essential 8 framework	Self-reported, objective physical measurements CVH assessment timing not specified
Sánchez-Díaz Carola, 2024, Journal of the American College of Cardiology (JACC) – Cardio-Oncology [32]	United States	Prospective cohort	Not defined	*N* = 713 Breast cancer; Female; Age: 55.4 ± 10.8 Black (African American); 35% less than high school graduate; 35% household income $25,000-$69,999	Data from Women’s Circle of Health Follow-Up Study, a population-based study of Black breast cancer survivors in New Jersey. Participants were identified through rapid case ascertainment in 10 New Jersey counties by the New Jersey State Cancer Registry, cancer survivorship status was self-reported	Both AHA’s Life’s Simple 7 & Life’s Essential 8 frameworks	Baseline interview approximately 10 months after diagnosis, annual follow-up interviews conducted through home visits (including anthropometric measurements and blood tests) CVH assessment approximately 24 months after diagnosis
Satti, 2024, JACC – Cardio-Oncology [9]	United States	Cross-sectional analysis	Not defined	*N* = 8,254 Mixed cancer types; 45.5% male; Age: 62.4% ≥ 65 years 89.7% White; 34.6% less than high school education; Income not reported	Data from NHIS (2013–2017), cancer survivorship status was self-reported	AHA’s Life’s Essential 8 framework (without dietary data)	Self-reported CVH assessment timing not specified
Wadden, 2024, JACC – Cardio-Oncology [33]	United States	Prospective cohort	Not defined, Participant reports of breast cancer were verified by centrally trained adjudicators	*N* = 7,165 Breast cancer; Female; Age (at diagnosis): 70.1 ± 7.5 89.2% White; 49.2% high-school to bachelor’s; 42.1% income $35,000-$74,999	Data from Women’s Health Initiative (WHI), a prospective cohort study of postmenopausal women. The WHI study enrolled a total of 161,808 postmenopausal women 50 to 79 years of age at 40 clinical centers from 1993 to 1998 in the United States	AHA’s Life’s Essential 8 framework	Self-reported, measured during in-person at clinic visits CVH assessment timing differs among sub-groups within the cohort (all metrics were measured at enrollment, then measured yearly, or every three years) The timepoint closest to but prior to breast cancer diagnosis was used
Weaver, 2024, BMC-Cancer [25]	United States	Cross-sectional analysis (part of the AH-HA study)	At least three months post-potentially curative treatment for cancer	*N* = 502 Mixed cancer type (79.7% breast cancer); 95.6% female; Age: 51.2% 40–65 years; 86.3% non-Hispanic White; 44.4% college degree or more; Income not reported	Baseline data from an ongoing NCI Community Oncology Research Program trial of the AH-HA study	AHA’s Life’s Simple 7 framework	Self-reported, medical record CVH assessed post-diagnosis and treatment

### How was CVH conceptualized and measured in the included studies?

Regarding the conceptualization of CVH in included studies, more than half (*n* = 14) of the studies employed the AHA’s LS7 or LE8 frameworks to assess CVH (23%, *n* = 5 used LS7 [22–26], 41%, *n* = 9 used LE8 [9, 27–34]). Eight studies (36%) used other conceptualizations of CVH or cardiovascular risks [35–42] (Table 4). Most studies collected CVH data after the diagnosis and completion of cancer treatment, with specific time frames ranging from as early as three months post-treatment to more than three years after diagnosis. Two studies [33, 39] examined pre-diagnosis CVH and associated survivorship outcomes. A majority of the studies using existing databases did not specify the exact timing of the CVH assessment relative to the cancer diagnosis.

**Table 4:** Methods of measurement used for assessing the physical activity, diet quality, nicotine exposure, and sleep health components of ideal CVH metrics (*N* = 22)

First author’s last name, year	CVH Measurement	CVH metrics	Range, categories, composite score, scoring methods	Physical activity	Diet quality	Nicotine exposure	Sleep health
Enright, 2010 [37]	Five key modifiable cardiac risk factors	Blood pressure, weight, cholesterol, smoking status, exercise	The risk factor was defined as present (or uncontrolled) if it did not meet the American Heart Association (AHA)/American College of Cardiology (ACC) guidelines The risk factors were assessed individually and as a global cardiac risk score (range: 0–5), which was defined as the total number of risk factors that did not meet the targets	Self-reported, AHA/ACC target: 30 min of moderate exercise, three times per week	Not assessed	Self-reported, AHA/ACC target: Non-smoker, ex-smoker	Not assessed
Hawkes, 2011 [38]	Five lifestyle factors	Physical activity, television viewing, smoking, alcohol consumption, BMI^a^	No composite score	Assessed using standardized instrument: Active Australia Survey Self-reported amount of time (minutes) they spent, in a usual week over the past month: Walking for transport or recreation; in other moderate-intensity physical activity, and in vigorous-intensity physical activity	Not assessed (Self-reported alcohol consumption: estimation of the average number of standard alcoholic drinks they consumed per week, based on the current Australian guidelines for minimizing harm from alcohol consumption)	Self-reported smoking status: current smokers; former smokers; or never smokers	Not assessed
Weaver, 2013 [42]	Six CVD risk factors	Smoking, BMI, physical inactivity, hypercholesterolemia, hypertension, diabetes	Composite score: the sum of the number of CVD risk factors (current cigarette smoking, no moderate or vigorous physical activity in the past month, overweight or obese body mass index, ever diagnosis with hypertension, and ever diagnosis with diabetes) (range: 0–5)	Self-reported number of times per week and the minutes per episode of moderate and vigorous physical activities Three physical activity groups: meeting American College of Sports Medicine (ACSM)/ AHA’s PA guidelines for adults (≥ 150 min of moderate-intensity or 60 min of vigorous-intensity physical activity per week), some physical activity, but below guideline level, or no physical activity	Not assessed	Self-reported smoking status: defined as current (daily or “some days a month”, former (at least 100 lifetime cigarettes but not currently smoking), and never (less than 100 lifetime cigarettes)	Not assessed
Pelser, 2014 [39]	Five lifestyle factors	Healthy diet, BMI, physical activity, alcohol consumption, smoking	Composite lifestyle score: each lifestyle variable was dichotomized and assigned one point for meeting the recommendation and zero points for not meeting it, ranging from 0 (worst score) to 5 (best score)	Self-reported frequency of engaging in 20 min activity that resulted in increased breathing, heart rate, or perspiration Categorized into five levels (never or rarely, 1–3 times/ month, 1–2 times/week, 3–5 times/week, and!5 times/ week)	Self-reported: a 124-item food frequency questionnaire, in which participants reported the frequency of food and beverage consumption for the previous 12 months Dietary quality was determined by applying the Healthy Eating Index 2005 (HEI-2005), which assesses conformance to the 2005 Dietary Guidelines for Americans (scores range from zero to 100 points)	Self-reported smoking status and time since quitting for former smokers Smoking history was divided into 4 categories (never smoked, quit 10 years ago, quit 1–9 years ago, quit < 1 year ago, or current smoker)	Not assessed
Song, 2020 [41]	Three health behaviors Three major CVD risk factors	Health behavior factors: smoking, physical activity, diet intake (based on the US Preventive Services Task Force Recommendation) CVD risk factors: hypertension, high cholesterol, diabetes	No composite score	Self-reported using the question: “currently spend half hour or more in moderate to vigorous physical activity at least five times a week” answered by yes or no	Not assessed since Medical Expenditure Panel Survey (MEPS) did not include questions on dietary intake, BMI was used as a crude proxy of diet quality	Self-reported smoking status: question “currently smoke” answered by yes or no	Not assessed
Foraker, 2021 [22]	LS7	CVH factors: cholesterol, blood pressure, glucose/hemoglobin A1C CVH behaviors: BMI, smoking, diet, physical activity	CVH score is automatically calculated by the AH-HA tool once the patients enter self-reported data on the 7 CVH factors A score of 100% = best CVH A score of 0% = worst CVH	Self-reported and from medical record Followed the AHA’s LS7 evaluation guideline [5]			
Weaver, 2021 [23]	LS7	Health behaviors: smoking status, BMI, physical activity, diet Health factors: cholesterol level, blood pressure, blood glucose level	CVH score is automatically calculated by the AH-HA tool once the patients enter self-reported data on the 7 CVH factors A score of 100% = best CVH A score of 0% = worst CVH	Self-reported and from medical record Followed the AHA’s LS7 evaluation guideline [5]			
Coughlin, 2022 [36]	Five risky health behaviors	Current smoking status, alcohol consumption, consumption of fruits/vegetables per day (diet quality), physical activity, body weight	No composite score	Self-reported physical activity: Participants were determined to be physically inactive if no physical activity or exercise other than regular job was reported during the past 30 days	Self-reported diet quality: No consumption of fruits/vegetables refers to consumption of fruits/vegetables < 1 time per day (Self-reported alcohol consumption: heavy alcohol use was defined as an average intake of 14 drinks per week for men and 7 drinks per week for women)	Self-reported smoking status: current smokers were defined as respondents who smoked at least 100 cigarettes in their lifetime and now smoke some days or every day	Not assessed
Kaneko, 2022 [26]	LS7 (LE8 was also calculated and used for supplementary analysis)	Smoking, BMI, physical activity, dietary habits, blood pressure, fasting glucose level, and total cholesterol level	No composite score; each LS7 metric was categorized into “ideal” or “non-ideal”	Self-reported, ideal physical activity was defined as 30 min of exercise at least twice a week or ≥ 1 h of walking per day	Self-reported, ideal eating habits were defined as skipping breakfast < 3 times per week	Self-reported, ideal smoking status was defined as not smoking (never smoked or prior smoker), and current smoker was defined as smoking ≥ 100 cigarettes in a lifetime or smoking duration ≥ 6 months	Self-reported, ideal sleep was defined as answering “Yes” the following question ‘Do you have a good rest with sleep?’
Chan, 2023 [27]	LE8 without dietary data	Hypertension, diabetes mellitus, dyslipidaemia, physical inactivity, inappropriate sleep duration, smoking, obesity	Each CVH domain equals one-point (if the condition exists); higher scores indicated poorer CVH	Physical inactive if not engaging in ≥ 75 min/week of vigorous-intensity activity, ≥ 150 min/week moderate‐intensity activity or combination, or a total combination of ≥ 150 min per week of moderate/vigorous‐intensity aerobic physical activity	Not assessed Not included as the NHIS contains no dietary data	Self-reported current smoking status	Not assessed
Connor, 2023 [35]	Five behavioral/ lifestyle factors	BMI (at breast cancer diagnosis, and at age 25 years), smoking status, alcohol consumption, diet/nutrition, physical activity	No composite score	Self-reported number of times per week and the minutes per episode of moderate and vigorous physical activities and strength training Following the ACSM guideline: weekly aerobic activities recommendations were met if participants reported at least 150 min/week of moderate-intensity or at least 75 min/week of vigorous-intensity activity on three or more days/week Weekly strength training recommendations were met if the participant reported strength training activities of at least moderate-intensity on at least two days/week	Assessment method was not described, but reported as five categories: Excellent, very good, good, fair, poor	Self-reported with four questions: Age began smoking cigarettes, Time since quitting smoking cigarettes (years), When quit, # of cigarettes smoked daily, Past 30 days on the days smoked, how many cigarettes smoked	Not assessed
DeMari, 2023 [24]	LS7	Health behaviors: smoking status, BMI, physical activity, diet Health factors: cholesterol level, blood pressure, blood glucose level	2 points: ideal metric, 1 point: intermediate metric, 0 point: poor metric The sum of these values is then divided by the total possible number of points (maximum of 14) Total composite score = 0–100 High CVH = 73–100 Moderate CVH = 50–72 Low CVH = 0–49	Self-reported Ideal health: ≥150 min/week moderate or ≥ 75 min/week vigorous or ≥ 150 min/week moderate and vigorous	Self-reported Ideal health: meeting 4–5 following components Consumed ≥ 4.5 cups/day of fruits and vegetables, ≥ 2 3.5-oz servings/week of fish, 3 1-oz equivalent servings/day of fiber-rich whole grains, restricted their diet to < 1500 mg/day of sodium ≤ 450 kcal/week of sugar-sweetened beverages	Self-reported Ideal health: Never smoked or quit > 12 months	Not assessed
Peng, 2023 [40]	“Healthy lifestyle score” with five modifiable lifestyle factors	BMI, smoking, alcohol drinking, dietary habits, physical activity	A healthy lifestyle score was defined according to the number of low-risk lifestyle factors, ranging from 0 to 5, with a higher score indicating a healthier lifestyle. The healthy lifestyle score was subsequently divided into four groups (0–1 points, 2 points, 3 points, and 4–5 points)	Self-reported, Low-risk group for physical activity included those engaged in at least 150 min of moderate physical activity or at least 75 min of vigorous physical activity per week	Self-reported, Dietary score generated using the following five dietary habits: consumption of vegetables ≥ 4 servings/day, fruits ≥ 3 servings/day, fish ≥ 2 servings/week, processed meat ≤ 2 servings/week, unprocessed red meat ≤ 1 serving/week A dietary score ≥ 4 was considered a healthy diet (daily alcohol consumption < 14 g were defined as low-risk)	Self-reported, No current smoking was considered a low-risk group	Not assessed
Zhang, 2023 [34]	LE8	4 health behaviors: diet, physical activity, smoking, and sleep 4 healthy factors: BMI, non-high-density lipoprotein (HDL) cholesterol, blood glucose, blood pressure	The aggregate score is scaled from 0 to 100 points, calculated as the unweighted average of 8 component metric scores	Supplementary material not assessable	Self-reported, assessed according to the status of salt intake, tea consumption, and fatty food intake in this study	Supplementary material not assessable	Supplementary material not assessable
Fan, 2024 [28]	LE8	4 health-related behaviors: diet, physical activity, nicotine exposure, and sleep 4 health-related factors: BMI, non-HDL cholesterol, blood glucose, blood pressure	Each CVH metric’s score ranged 0 to 100, scores are used to calculate the overall LE 8 score, with varying weights assigned to different health behaviors and factors in assessing individual CVH importance according to AHA’s LE8 framework The total LE8 score is the arithmetic mean of the eight metrics High CVH = 80–100 Moderate CVH = 60–79 Low CVH = 0–59	Self-reported weekly minutes of moderate or vigorous physical activity, categorized into different levels	Self-reported, assessed based on the 2015 Healthy Eating Index (HEI), incorporating participants’ dietary intake and United States Department of Agriculture food pattern data	Self-reported, nicotine exposure classification includes smokers and non-smokers, with varying scores based on smoking history and environmental exposure	Self-reported nightly sleep duration
Lefferts, 2024 [29]	LE8	4 ideal health behaviors: diet, physical activity, nicotine exposure, sleep 4 ideal health factors: BMI, blood lipids, glucose, blood pressure	Total CVH score was calculated as the average of all 8 individual CVH metric score High CVH = 80–100 Moderate CVH = 60–79 Low CVH = 0–59	Self-reported, assessed using standardized instrument: International Physical Activity Questionnaire Frequency per week and duration of each session were recorded for both moderate and vigorous physical activity	Self-reported, assessed using standardized instrument: Dietary Screening Tool Scores on the Dietary Screening Tool range from 0 to 100, with 5 bonus points available for the use of a dietary supplement	Self-reported, assessed from 3 self-report questions: 1. Have you ever smoked at least 100 cigarettes in your lifetime? 2. Do you currently smoke cigarettes? 3. How many years ago did you stop smoking cigarettes? (Data were not available for secondhand smoke exposure)	Self-reported, assessed using standardized instrument: Pittsburgh Sleep Quality Index Self-reported duration of sleep per day were classified as < 4, 4 to < 5, 5 to < 6, 6 to < 7, 7 to < 9, and 9 to < 10 h
Lopez-Bueno, 2024 [30]	LE8	4 health behaviors: nicotine exposure, physical activity, diet, sleep 4 health factors: BMI, blood glucose levels, blood lipid levels, blood pressure	The LE8 score is calculated as the mean value of these 8 components graded on a scale 0 to 100 High CVH = ≥ 80 Moderate CVH = ≥ 50–<80 Low CVH = < 50	Not specified, followed the AHA’s LE8 evaluation guideline [6]			
Qiu, 2024 [31]	LE8	Diet, physical activity, exposure to tobacco or nicotine, sleep quality, BMI, levels of non-high-density lipoprotein cholesterol (non-HDL), blood glucose, blood pressure	High CVH = ≥ 80 Moderate CVH = ≥ 50–<80 Low CVH = < 50	Self-reported minutes of moderate or vigorous physical activity per week	Self-reported, assessed using the Dietary Approaches to Stop Hypertension (DASH) diet score, calculated from the average values of dietary components gathered through two non-consecutive 24-hour dietary recalls at the outset	Self-reported tobacco/nicotine exposure	Self-reported sleep duration
Sánchez-Díaz Carola, 2024 [32]	LE7 and LE8	BMI, diet, smoking, physical activity, blood pressure, total cholesterol, blood glucose, sleep	The LS7 scores range from 0 to 14, and the LS7 plus sleep scores range from 0 to 16, higher scores indicate better CVH	Self-reported, assessed using standardized instrument: Godin Leisure-Time Exercise Questionnaire	Self-reported, dietary intake over the past year was assessed using an 18-item food frequency questionnaire	Self-reported, cigarette smoking history since diagnosis was assessed during the interviewer-administered interview	Self-reported, average sleep hours over the past month assessed using the Pittsburgh Sleep Quality Index questionnaire
Satti, 2024 [9]	LE8 without dietary data	7 binary domains/risk factors: hypertension, diabetes mellitus, hypercholesterolemia, current smoking, physical activity, inappropriate sleep, obesity	Each of the 7 CVH domains was coded as 0 (absence of a risk factor) or 1 (presence of a risk factor), with a maximum composite CVH score of 7	Self-reported, insufficient physical activity was defined as not engaging in 75 min/week of vigorous exercise, 150 min/week of moderate intensity exercise or combination, or a total combination of 150 min/week of moderate intensity/ vigorous exercise	Not assessed, the NHIS does not include detailed dietary data	Self-reported current smoking status	Self-reported, inappropriate sleep duration was defined as < 6 h or > 10 h of sleep on average per night
Wadden, 2024 [33]	LE8	Diet, physical activity, nicotine exposure, sleep health, BMI, blood lipids, blood glucose, blood pressure	Each metric has a total possible score of 100, and the unweighted sub scores were added and divided by 8 for a final LE8 score that ranged from 0 to 100, with higher scores indicating a more favorable health state High CVH = 80–100 Moderate CVH = 50–79 Low CVH = 0–49	Self-reported minutes of moderate or vigorous PA per week, followed the AHA’s LE8 evaluation guideline [6]	Self-reported, assessed using standardized instrument: Health Eating Index-2015 Total Score calculated from Food Frequency Questionnaires	Self-reported use of cigarettes (never smoker, previous smoker, current smoker)	Self-reported average hours of sleep per night
Weaver, 2024 [25]	LS7	Health behaviors: smoking status, BMI, physical activity, diet Health factors: cholesterol level, blood pressure, blood glucose level	Each metric was scored as ideal, intermediate, poor, or missing/unknown according to the AHA LS7 framework	Followed the AHA’s LS7 evaluation guideline. Self-reported and most recent value abstracted from the electronic health records [5]			

Notes:

^a^ The AHA’s Life’s Essential 8 (LE8) categorizes BMI/ bodyweight as a health factor, reflecting a distinction between behavioral and health metrics in the framework. However, some studies in this review classified BMI or body weight as a behavioral factor, diverging from the LE8’s classification. This discrepancy is rooted in the original Life’s Simple 7 (LS7) framework, which categorized BMI as a behavioral metric. Such inconsistencies highlight variations in how CVH metrics are defined across studies, complicating comparisons and potentially influencing interpretations in CVH research among cancer survivors

As shown on Table 4, a few studies followed the LE8 framework but had missing diet quality data [9, 27], due to data not being available in the dataset used in the analyses. The eight studies that did not follow the AHA’s framework used alternative lifestyle behaviors factors such as television viewing and alcohol consumption to assess CVH [38]. Among studies that followed the LS7 or LE8 frameworks, some studies adhered to the AHA’s standardized guidelines when calculating composite scores for ideal CVH [24, 30], while a few studies employed alternative scoring methods driven primarily by data availability [26, 27]. Additionally, discrepancies existed among studies regarding the thresholds used to categorize CVH as ideal, moderate, or poor [28, 29]. Finally, four out of eight studies that did not follow the AHA’s framework did not have a composite score for CVH [35, 36, 38, 41]. 

### How were CVH metrics measured in the included studies?

The four health metrics (i.e., BMI, blood lipids, blood glucose, and blood pressure) were measured by standardized laboratory testing procedures during visits to a health care provider or were retrieved from electronic medical records (Table 3). One study required cancer survivors to self-report values of these metrics and compared them to medical records [25]. On the other hand, the measurement methods of the four lifestyle behavior metrics (i.e., physical activity, diet quality, nicotine exposure, sleep health) were highly heterogenous. The measurement methods of the four lifestyle behavior metrics among the included articles are depicted in Table 4.

Assessment methods for each lifestyle behavior factor were highly variable and were mostly self-reported. Physical activity was assessed by self-reported amount of time spent on different levels of intensity of activities per day, week, or month. Various standardized and validated questionnaires such as the Active Australia Survey [43], the International Physical Activity Questionnaire [44], and the Godin Leisure-Time Exercise Questionnaire [45] were also used. None of the included studies used an assessment of the volume, duration, intensity, and pattern of physical activities which involved digital trackers. More than half of the studies assessing physical activity reported data on physical activity intensity (i.e., light, moderate, vigorous activities).

Diet quality measurements were highly heterogeneous. The AHA’s guideline recommended assessing diet quality using self-reported daily intake of a “Dietary Approaches to Stop Hypertension” (DASH)-style diet pattern with eight components, including high intake of fruits, vegetable, nuts and legumes, whole grains, low fat dairy, and low intake of sodium, red and processed meats, and sweetened beverages [6]. Most studies reported estimated salt intake, estimated fatty food intake, or reported intake of fruits and vegetables only. Six studies lacked dietary data in their data source, thus omitting this factor in their calculation of the CVH composite score. A few studies used standardized questionaries such as the Healthy Eating Index [46] and the dietary screening tool [47] or using dietary recalls. Unique among the included studies, a study conducted in Japan defined ideal diet habit as “skipping breakfast less than 3 times per week” [26]. 

In a majority of studies, nicotine exposure was self-reported in a dichotomous fashion (currently smoking vs. not smoking), with only a few studies assessing the duration or frequency and types of tobacco products used. One study assessed environmental exposure of nicotine, but without giving details about the types [28]. None of the included studies assessed secondhand smoke exposure. Finally, ten studies assessed sleep health, the newly proposed eighth factor of ideal CVH by the AHA [6]. Four studies assessed sleep health through self-report nightly sleep duration, and two used the Pittsburgh Sleep Quality Index [48]. One study used a “Yes” or “No” statement “Do you have a good rest with sleep?”

### What were the findings of the included studies?

The included studies’ objectives and main findings are outlined in Table 5. The findings highlight a range of factors associated with CVH in cancer survivors. Overall, worse CHV among cancer survivors was associated with higher risks of adverse CVD outcomes (seven studies [26, 29, 33, 34, 36, 38, 40]) as well as cancer and all-cause mortality (four studies [28, 30, 33, 39]). Studies showed that modifiable lifestyle behavior factors such as smoking, inactivity, and obesity were more prevalent among survivors when comparing to the general population, with specific associations to demographic characteristics such as age, gender, race/ethnicity, and other social determinants of health factors (e.g., neighbor environments and geographic locations). For instance, one cross-sectional study indicated that in comparison to the general population, cancer survivors who have multiple CVD risks factors had higher rates of cardiovascular conditions, including myocardial infarction and coronary heart disease, particularly among Black and Hispanic groups [36]. Some inconsistent findings were reported. For instance, older survivors and those from minority racial/ethnic groups had higher CVD risks [37], while one study showed association with worse CVH in younger survivors and women [9]. 

**Table 5:** Study objectives, factors associated with ideal CVH among cancer survivors, and main findings as a function of study designs (*N* = 22)

First author’s last name, year	Study Objective(s)	Factors or variables associated with CVH	Main findings/ Comparison to the general population (if applicable)
**Cross-sectional studies (** ***n*** ** = 9)**			
Enright, 2010 [37]	Determined the prevalence of multiple uncontrolled modifiable cardiac risk factors and identified variables associated with poor control of cardiac risk factors among cancer survivors and matched controls	Age	**Cancer survivors**: • 91.5% had multiple risk factors, 35.2% at high cardiac risk • More likely to be smokers • Among survivors, only age was associated with cardiac risk factors, with older survivors significantly more likely to have poor control than younger survivors
			**General population**: • 89.9% had multiple risk factors, 32.6% at high cardiac risk • More likely to be overweight
Weaver, 2013 [42]	Assessed the prevalence of CVD risk factors among long-term cancer survivors and compare results to survey data from the general population in the same geographic region	Race/ ethnicity	**Cancer survivors**: • More likely to have CVD risk factors • Compared to white, non-Hispanic survivors, Hispanic and African American (but not Asian survivors) reported significantly more risk factors
			**General population**: • More likely to be smokers
Song, 2020 [41]	Examined the prevalences of CVD, CVD risk factors and health behaviors among cancer survivor-spouse dyads, assessed how these prevalences differ by role (survivor vs. spouse) and gender, and reported congruences in health behaviors between survivors and their spouses	Gender and role (survivor vs. spouse) differences	Gender and role differences were significantly related to the prevalence of CVD, CVD risk factors, and health behaviors among survivors and spouses. The odds of males currently non-smoking was 0.81 and the odds of males engaging in physical activity was 1.21 compared to females. The male survivors had the highest BMI, and female survivors had the lowest BMI 39–88% of survivors and spouses were congruent in their current smoking status, physical activity engagement/disengagement, and BMI
Coughlin, 2022 [36]	Examined CV conditions and risk factors among cancer survivors Compared CV conditions and risk factors among cancer survivors and men and women without a history of cancer	5 self-reported chronic health conditions: myocardial infarction, coronary heart disease (CHD), diabetes, hypertension, high cholesterol Socioeconomic status (i.e., race/ ethnicity)	**Cancer survivors**: • More likely to have multiple risk factors (cigarette smoking, physical inactivity, and obesity) • Have a higher prevalence of heart attack, CHD, diabetes, and hypertension • Higher prevalence of heart attack, CHD, and diabetes among Black and Hispanic cancer survivors
			**General population**: • More likely to be heavy drinkers or to not consume fruits and vegetables
Chan, 2023 [27]	Investigated the relationship between psychological distress and CVH amongst cancer survivors	Psychological distress was measured by the six-item Kessler scale	**Cancer survivors**: • Severe psychological distress (SPD) was independently associated with worse CVH • Strong relationship between SPD and known CVD • Association between SPD and CVH was stronger in those who were younger or female
			**General population**: • SPD was independently associated with worse CVH
Conner, 2023 [35]	Described the burden of obesity, comorbidity, and behavioral factors associated with CVD risk among a sample of Black breast cancer survivors in Maryland, US, and explored differences by county to determine if potential societal differences may exist	Geographic locations (county)	Only 28% of the survivors reported meeting weekly exercise recommendations. While 70% were never smokers, most ever smokers resided in Baltimore City/Baltimore County
Lefferts, 2024 [29]	Investigated the association between adherence to the AHA’s LE8 and arterial stiffness in older adult survivors of cancer	Arterial Stiffness assessed using carotid-femoral pulse wave velocity	Cancer survivors with greater adherence to the AHA’s LE8 for CVH have lower prevalence of high arterial stiffness
Qui, 2024 [31]	Explored the connection between LE8 scores and frailty levels in cancer survivors across the United State	Frailty (i.e., result of cumulative cellular damage, subsequently leading to a decline in organ system function and a reduced ability to restore homeostasis after stress events), measured using the frailty index	Increased LE8 level was closely associated with a reduced odds ratio of frailty among cancer survivors
Satti, 2024 [9]	Investigated associations between the social determinants of health (SDOH) and CVH of adult cancer survivors	Social determinants of health, assessed using six domains: economic stability, neighborhood, community and social context, food poverty, education, access to health care	Worse SDOH was associated with worse CVH, with significantly stronger associations in younger participants or women
**Prospective cohort studies (n = 6)**			
Hawkes, 2011 [38]	Assessed self-reported lifetime prevalence of CVD among colorectal cancer survivors, and examined the cross-sectional and prospective associations of lifestyle factors with co-morbid CVD	Six CVD categories: hypercholesterolemia, hypertension, diabetes, heart failure, kidney disease, and ischemic heart disease (IHD)	Co-morbid CVD prevalence at 5 months post-diagnosis was 59%, and 16% of participants with no known CVD at baseline reported de novo CVD by 36 months BMI was the strongest correlate of co-morbid CVD, with over-weight/obese males and females more likely to suffer from hypercholesterolemia, hypertension, diabetes and IHD Obesity at baseline predicted de novo hypertension and de novo diabetes
Peng, 2023 [40]	Investigated the association between healthy lifestyle factors and CVD risk among female breast cancer survivors from a large population-based cohort study (UK Biobank) Explored whether healthy lifestyle patterns might modify the association between the polygenetic risk scores (PRS) of CVD and the risk of incident CVD	PRS derived for coronary heart disease (CHD), ischemic stroke (IS), and heart failure (HF)	Participants with 4–5 healthy lifestyle components were associated with a decreased risk of incident CVD, CHD, IS, and HF, compared with those with 0–1 lifestyle component Evidence for the genetic–lifestyle interaction was observed for CHD and HF. Among participants at high genetic risk, a healthy lifestyle was associated with a lower risk of CHD, IS and HF
Zhang, 2023 [34]	Investigated the associations between CVH assessed by the LE8 with risk of incident atherosclerotic CVD (ASCVD) and ASCVD-related mortality amongst cancer patients	Incident atherosclerotic cardiovascular disease (ASCVD): myocardial infarction (MI), IS, HF, coronary revascularization	Compared to patients in Tertile 1 of LE8 (33.3% of the sample with the lowest LE8 score), cancer survivors in Tertile 3 had 46% lower risks of developing composite ASCVD events and a 40% lower ASCVD related mortality risk
Lopez-Bueno, 2024 [30]	Examined the dose-response association of CVH with all-cause, CVD, and cancer mortality among US adult cancer survivor	All-cause, cardiovascular and cancer deaths: ascertained using a probabilistic record matching method with the National Death Index records	Inverse relationship was found between higher LE8 and risk of death from all cause, an inverse curvilinear relationship between higher LE8 and the risk for CVD death, and a non-significant association between higher LE8 and the risk of cancer death
Sánchez-Díaz Carola, 2024 [32]	Characterized the neighborhood archetypes where black breast cancer survivors resided at diagnosis and evaluated their associations with CVH	Four neighborhood archetypes: Mostly Culturally Black and Hispanic/Mixed Land Use archetype, Culturally Diverse/Mixed Land Use archetype, mostly Culturally Black/Green-centric archetype, Culturally Diverse/Green-centric archetype	Women in the “Mostly Culturally Black and Hispanic/Mixed Land Use” archetype showed the lowest CVH scores Compared to this archetype, black breast cancer survivors in the “Culturally Diverse/Mixed Land Use“ archetype were nearly 3 times as likely to have optimal CVH, with a stronger association observed in younger or premenopausal women
Wadden, 2024 [33]	Evaluated the incidence of CVD in relation to the LE8 score measured prior to diagnosis of breast cancer	Composite of incident CVD events, which included coronary heart disease, defined as myocardial infarction along with coronary revascularization, CVD death	Higher LE8 scores prior to diagnosis were associated with a lower risk of incident CVD among women with breast cancer in the United States
**Retrospective cohort studies (n = 3)**			
Pelser, 2024 [39]	Examined the relationship of pre-diagnosis lifestyle factors, alone and in combination, on five-year all-cause, colorectal cancer–specific, and CVD mortality among colon and rectal cancer survivors	Total mortality, all-cause mortality, colorectal cancer mortality, all death and CVD death	In colon cancer survivors, smokers had increased risk of total mortality and colorectal cancer mortality. Obese individuals had increased risk of all death and CVD death. In rectal cancer survivors, individuals in the highest quintile of diet quality scores had reduced all-cause mortality Higher combined lifestyle scores were associated with a 46% lower risk of total mortality
Kaneko, 2022 [26]	Aimed to clarify the association LS7 with incident CVD among cancer survivors Analyzed the relationship between the change in LS7 and the subsequent CVD risk	Composite CVD outcome: MI, angina pectoris, stroke, HF	Risk of CVD events increased with a greater number of non-ideal LS7: the hazard ratio per 1-point increase in non-ideal Life’s Simple 7 was 1.15; a 1-point increase in non-ideal LS7 over 1 year was associated with subsequent CVD risk among cancer survivors
Fan, 2024 [28]	Investigated LE8’s associations with mortality outcomes in cancer survivors	All-cause, cancer-specific and non-cancer mortality	High CVH was associated with lower hazard ratios for all-cause, cancer-specific and non-cancer mortality vs. low CVH Cumulative mortality rates increased during follow-up, more so in the low CVH group Subgroup analysis revealed significant LE8 interactions with age or Poverty Income Ratio (PIR) for all-cause mortality
**First author**,** year**	**Objective(s) and main findings**		
**Assessment tool development: The Automated Heart-Health Assessment (AH-HA) tool (n = 4)**			
Foraker, 2021 [22]	Described the Automated Heart-Health Assessment (AH-HA) study protocol. This study assessed the effect of a clinical decision support tool for cancer survivors on CVH discussions, referrals, completed visits with primary care providers and cardiologists, and control of modifiable CVH factors and behaviors		
Weaver, 2021 [23]	Evaluated breast cancer survivors’ awareness of cardiovascular risk factors and examined the usability of a novel electronic health record enabled CVH tool from the perspective of both breast cancer survivors and oncology providers. The study’s findings suggested that the AH-HA tool is well-accepted in oncology practices, with both providers and breast cancer survivors likely valuing its integration into survivorship care		
DeMari, 2023 [24]	Assessed endometrial cancer survivors’ perspectives on addressing CVD risk during oncology care, the results suggested that survivors were receptive to discussions about cardiovascular risk during routine oncology care		
Weaver, 2024 [25]	Assessed cancer survivors’ CVH profiles, compared self-reported and electronic health records-based categorization of CVH factors, described perceptions regarding addressing CVH during oncology encounters. The study revealed that over 50% of survivors had consistent categorizations between electronic health records and self-reports for smoking, BMI, and blood pressure, while information on cholesterol, glucose, and A1C was often missing or unknown to the survivors. The authors concluded that tools that facilitate CVH discussions can help bridge knowledge gaps and are likely to be well-received by survivors in community settings		

One study examined the association between neighborhood environment and CVH among black breast cancer survivors and reported that survivors in culturally diverse areas had better CVH outcomes compared to those in less diverse areas [32]. Another study reported an association between severe psychological distress and worse CVH among cancer survivors [27]. Further, a study reported that higher scores on ideal CVH was associated with reduced frailty among cancer survivors [31]. Overall, the studies emphasize the complex and multifaceted nature of CVH among cancer survivors. Finally, studies on the Automated Heart-Health Assessment (AH-HA) tool [23–25] suggested strong acceptance of an electronic health record-enabled CVH assessment tool, which improved awareness and discussions around CVH in oncology care, signaling a valuable approach for future survivorship care integration.

## Discussion

This scoping review highlights a growing number of studies examining ideal CVH in cancer survivorship literature, emphasizing the need to promote its adoption and maintenance among cancer survivors. Notably, the majority of included studies were published in or after 2020, with a significant increase in publications following the introduction of LE8 after 2022 [6]. This trend aligns with the broader rise in interest in cancer survivorship research, driven by improved survival rates and the expanding population of cancer survivors. Moreover, over the past two decades, there has been a significant shift in survivorship care, moving from an exclusive focus on acute cancer treatment to prioritizing tertiary prevention of long-term treatment side effects and promoting sustained health and well-being [49]. 

Our scoping review uncovers several gaps in the literature on ideal CVH among cancer survivors, highlighting areas for future investigation. First, the National Cancer Institute (NCI) defines cancer survivors as individuals with cancer from diagnosis to the end of life [17]. However, most studies included in this review focused on CVH metrics post-treatment, with limited consideration of pre-treatment phases. Emerging literature on cancer prehabilitation suggests that the survivorship definition could encompass pre-treatment stages [50]. Notably, only two studies examined CVH before diagnosis and its associations with CVD incidence or mortality [33, 39], leaving a critical gap in understanding how early CVH metrics influence survivorship outcomes.

Another important finding of our scoping review is that researchers in the United States contributed to 82% of all included studies, while only four studies were conducted in other countries. This finding points to a lack of country diversity in current CVH literature. Race/ethnicity is another social determinant that warrants further study because CVH outcomes and risk factors can vary significantly across racial and ethnic groups due to differences in socioeconomic status, access to healthcare, lifestyle behaviors, and exposure to environmental stressors [51]. The underrepresentation of diverse populations in CVH studies means that the unique needs and risk profiles of racial and ethnic minorities may be overlooked, potentially leading to disparities in cardiovascular care and outcomes among cancer survivors. Future research should prioritize inclusive studies that account for factors such as socio-economic status, race, and ethnic diversity, ensuring that findings and interventions can be effectively applied to diverse populations.

Although some studies included in this review examined mixed cancer populations, few conducted analyses that were tailored to specific cancers, limiting the ability to determine whether CVH risks vary by cancer site and diagnosis. The lack of stratified analyses is particularly concerning given that different cancer types are associated with distinct treatment regimens, comorbidities, and risk factors that may differentially impact concurrent and subsequent CVH [52]. For instance, cancer survivors who were treated with cardiotoxic agents (e.g., anthracyclines, trastuzumab, radiation therapy), increasing the risks of myocardial dysfunction, vascular injury, and heart failure among survivors [53]. Additionally, the included studies did not specifically examine age-related considerations in CVH among cancer survivors, despite evidence that aging and cancer treatments independently contribute to increased CVD risk [54]. These gaps in research limit a comprehensive understanding of how CVH differs across cancer subtypes and different age groups. Future research should prioritize comparative analyses across cancer sites and age groups to better characterize the differential effects of treatment exposures and aging on CVH.

Most studies included in this review are cross-sectional, underscoring the absence of longitudinal and quasi-experimental research. Questions remain about whether the elevated cardiovascular risks observed among cancer survivors stem from pre-existing conditions, lifestyle behaviors, cancer treatments, or a combination of these factors. Identifying whether pre-diagnosis CVH factors contribute to cancer susceptibility or post-treatment CVD risk could clarify these pathways and support the development of tailored prevention strategies involving both lifestyle interventions and risk stratification.

Standardized, longitudinal data collection of CVH across the cancer continuum, including pre-treatment and survivorship phases, is essential. Developing an ongoing portrait of the CVH profile of cancer survivors at different life stages would provide critical insights into health trajectories in this population. Employing a standardized frameworks such as the AHA’s LE8 or LS7 would improve consistency in data collection, analysis, and comparability across studies. Addressing current inconsistencies in data sources, methodologies, and analytic approaches is critical to advancing research and optimizing health outcomes for cancer survivors.

Nevertheless, since the LS7 and LE8, frameworks were designed and validated mainly on data from US population, there is a need to examine their applicability in other jurisdictions to address stark differences in healthcare systems and sociocultural environments [5, 6]. Such replication and extension would also reinforce the added-value of this framework for improving CVH in the general population and cancer survivors globally. Future studies should therefore validate and adapt these frameworks to consider culturally specific determinants, including dietary patterns, healthcare infrastructure differences, and social determinants of health.

We also identified methodological heterogeneity in ideal CVH measurement, operationalization, and analysis that are worth noting. Although the AHA established clear guidelines for the definition of each metric, the measurement methods and definitions of CVH metrics (especially for lifestyle behaviors) remains highly variable, which complicates comparison across studies. Specifically, diet and physical activity are often assessed using different criteria than the ones proposed by the AHA. In many cases, there were only minor differences in the criteria that were used (e.g. some but not all dietary categories were assessed, or different thresholds for physical activity were used). However, some deviations from proposed guidelines were quite substantial.

For example, although the AHA’s LE8 framework recommends assessing diet using a comprehensive DASH-style eating pattern that includes eight specific components (see Table 1 and supplementary material in [6]), some studies diverge significantly [6]. A study conducted in Japan defined ideal eating habits simply as skipping breakfast fewer than three times per week [26], while a study conducted in China focused on assessing estimated salt, tea, and fatty food intake [34]. Further, there are also a lack of standardized measurement for second-hand smoke exposure and sleep health, which are newly added factors in the AHA’s LE8.

This methodological heterogeneity highlights the challenge of drawing cross-study comparisons and underscores the need for standardization in measuring and defining lifestyle factors within CVH research, as variations in operational definitions can lead to different conclusions regarding CVH among cancer survivors. A previous systematic review [8] has highlighted similar heterogeneity in behavioral CVH factor assessments. Since these variations likely reflect different constraints facing researchers who conduct large-scale epidemiologic studies, it is important for future work to determine whether these different operationalizations of ideal CVH are indeed commensurate with one another.

Further, we observed LE8 and LS7 composite score categorization discrepancies in this scoping review. For instance, some studies did not strictly adhere to the standardized LS7 scoring criteria, instead defining ideal, intermediate, and poor cardiovascular health categories based on the availability of data within their datasets [26]. Similarly, inconsistencies were noted in the application of LE8 scores across studies. The LE8 provides a standardized scoring system to classify CVH, with high CVH ranging from 80 to 100 points, moderate from 50 to 79 points, and low from 0 to 49 points [6]. However, some studies diverged from this operationalization by using alternative ranges, such as defining moderate CVH as 60–79 points and low CVH as 0–59 points [28, 29]. Reclassifying moderate or low CVH could either overestimate or underestimate the prevalence of low CVH in cancer survivors, potentially affecting the identification of at-risk populations. These variations create discrepancies that may influence the interpretation of CVH status in cancer survivors, as well as impact clinical decision-making and public health strategies based on these findings. Standardizing the use of AHA’s scoring thresholds across studies would enhance the reliability of comparisons and support meta-analytic approaches.

Despite the increasing focus on CVH within cancer survivorship research, our review identified critical gaps in understanding the role of social and environmental determinants in shaping CVH outcomes in this population [55, 56]. Evidence from the general population consistently shows that individuals with higher socioeconomic status exhibit better CVH metrics. For instance, recent systematic reviews show that more educated and wealthier individuals had higher ideal CVH metrics [7, 51]. Our scoping review identified limited attention to social and environmental determinants of ideal CVH within cancer survivor population, with only a few studies explicitly examining these factors and their association with CVH outcomes [9, 32]. Socioeconomic status (SES) significantly influences both cancer incidence and cardiovascular outcomes; individuals of lower SES frequently experience compounded vulnerabilities, including increased cancer risks due to occupational exposures, barriers to healthcare access, and higher prevalence of lifestyle-related cardiovascular risk factors [57, 58]. Additional mechanisms contributing to these disparities include reduced adherence to preventive and therapeutic guidelines, increased financial burden associated with cancer treatments, and chronic psychosocial stress, further exacerbating cardiovascular risks [59].

In addition, social and environmental contexts may critically shape post-diagnosis cancer outcomes and help predict individuals at the highest risk of cancer incidence. For instance, factors such as SES and neighborhood environments could influence the adoption of healthy lifestyle behaviors and the management of cardiovascular risk factors in cancer survivors both prior to and following diagnosis [60]. 

Collectively, these observations underscore the compounded CVH burden faced by cancer survivors and emphasize the pressing need for research that integrates social and environmental determinants into the understanding of CVH in this population. Addressing these gaps through targeted interventions, such as evidence-based clinical guidelines and population-level initiatives, could play a pivotal role in mitigating CVD risk. For instance, clinical approaches might include tailored consultations provided by oncology clinicians, while community-based programs could enhance access to resources that support healthier lifestyle behaviors.

Lastly, our review leads to some important clinical and public health implications. Integrating standardized CVH assessments into routine survivorship care is essential to systematically identifying and mitigating CVD risks among cancer survivors. Future efforts should focus on integrating CVH risk assessments into electronic health records using frameworks such as the LE8 and LS7 to streamline implementation and ensure continuous cardiovascular monitoring in survivorship care. Multidisciplinary collaboration among oncologists, cardiologists, nurses, rehabilitation specialists, and primary care providers can further facilitate early risk stratification, personalized lifestyle interventions, and timely initiation of evidence-based cardiovascular prevention strategies for cancer survivors in hospital setting and in communities.

### Study limitations

First, the use of AHA’s framework to conceptualize CVH risk could inherently limit the generalizability of our results to global populations. Second, we restricted our search to adult cancer survivors thus eschewing work on childhood cancer survivors and early/pre-diagnosis life-style behaviours affect CVH in cancer survivors. Further, we did not extract detailed longitudinal data on changes in CVH metrics over time. This limitation restricts our ability to assess the deterioration or improvement of CVH risk across different phases of cancer survivorship, which may be critical for understanding long-term cardiovascular outcomes and the impacts of specific interventions. Extracting detailed longitudinal data would have required a different methodological approach and additional resources, which were beyond the scope of this review.

## Conclusion

In summary, by mapping the existing evidence and highlighting areas where further investigation is needed, this review contributes to the advancement of knowledge in the field of cardio-oncology and supports the development of targeted interventions for the management of CVH in cancer survivors.

## Electronic supplementary material

Below is the link to the electronic supplementary material.

Supplementary Material 1

## Data Availability

The materials and data supporting the conclusions of this article are available in the Open Science Framework repository: https://doi.org/10.17605/OSF.IO/MXRWK.

## Abbreviations

AHA: American Heart Association
BMI: Body Mass Index
CVD: Cardiovascular Disease
CVH: Cardiovascular Health
LE8: Life’s Essential 8
LS7: Life’s Simple 7
SES: Socioeconomic Status
