# Supplementary material for: Investigating modifiable risk factors associated with ideal cardiovascular health among cancer survivors: a scoping review
Source: Cardiooncology. 2025 Mar 31;11:34. doi: 10.1186/s40959-025-00329-2 (PMC11956365; doi:10.1186/s40959-025-00329-2)
Supplement: Supplementary file 1 — Supplementary Material 1 [file 40959_2025_329_MOESM1_ESM.docx]

Table of Contents

[Supplemental Table 1: Search strategies from Cochrane Library (Search conducted on 21 August 2024) 2](#_Toc193268202)

[Supplemental Table 2: Data extraction instrument 4](#_Toc193268203)

## Supplemental Table 1: Search strategies from Cochrane Library (Search conducted on 21 August 2024)

| **#** | **Query** | **Results** |
| --- | --- | --- |
| 1 | MeSH descriptor: [Cancer Survivors] explode all trees | 1065 |
| 2 | (cancer survivor):ti,ab,kw | 3342 |
| 3 | (cancer survivorship):ti,ab,kw | 1209 |
| 4 | #1 or #2 or #3 | 4292 |
| 5 | MeSH descriptor: [Cardiovascular Diseases] explode all trees | 156811 |
| 6 | MeSH descriptor: [Cardiovascular Abnormalities] explode all trees | 3768 |
| 7 | ("cardiovascular disease"):ti,ab,kw | 28437 |
| 8 | ("cardiovascular risk"):ti,ab,kw | 19024 |
| 9 | ("cardiovascular risk score"):ti,ab,kw | 159 |
| 10 | ("heart disease"):ti,ab,kw | 24661 |
| 11 | (Heart Disease Risk Factors):ti,ab,kw | 11775 |
| 12 | (cardiovascular health):ti,ab,kw | 21556 |
| 13 | (cardiovascular outcome*):ti,ab,kw | 45617 |
| 14 | #5 or #6 or #7 or #8 or #9 or #10 or #11 or #12 or #13 | 213770 |
| 15 | MeSH descriptor: [Life Style] explode all trees | 8826 |
| 16 | (lifestyle behavio*r):ti,ab,kw | 7625 |
| 17 | ("risk factor"):ti,ab,kw | 40280 |
| 18 | (Modifiable Risk Factors):ti,ab,kw | 1921 |
| 19 | (Life's Essential 8):ti,ab,kw | 1251 |
| 20 | (Life's Simple 7):ti,ab,kw | 1621 |
| 21 | (diet*):ti,ab,kw | 119739 |
| 22 | MeSH descriptor: [Exercise] explode all trees | 39191 |
| 23 | (physical activit*):ti,ab,kw | 67885 |
| 24 | (exercise*):ti,ab,kw | 148977 |
| 25 | MeSH descriptor: [Health Behavior] explode all trees | 48157 |
| 26 | (Smoking Cessation):ti,ab,kw | 13283 |
| 27 | (smoking):ti,ab,kw | 40607 |
| 28 | (nicotine exposure):ti,ab,kw | 1096 |
| 29 | MeSH descriptor: [Body Weight] explode all trees | 40767 |
| 30 | ("body weight"):ti,ab,kw | 66780 |
| 31 | MeSH descriptor: [Body Mass Index] explode all trees | 14128 |
| 32 | ("body mass index"):ti,ab,kw | 52221 |
| 33 | (BMI):ti,ab,kw | 62584 |
| 34 | MeSH descriptor: [Sleep] explode all trees | 8877 |
| 35 | (sleep health):ti,ab,kw | 14241 |
| 36 | (Sleep Hygiene):ti,ab,kw | 1558 |
| 37 | MeSH descriptor: [Sleep Hygiene] explode all trees | 434 |
| 38 | #15 or #16 or #17 or #18 or #19 or #20 or #21 or #22 or #23 or #24 or #25 or #26 or #27 or #28 or #29 or #30 or #31 or #32 or #33 or #34 or #35 or #36 or #37 | 474628 |
| 39 | #4 and #14 and #38 | **204** |

## Supplemental Table 2: Data extraction instrument

| **Article #: (citation)** | | |
| --- | --- | --- |
| **Study Title** | | |
| **Study design** |  | **Notes** |
| **Article published year** |  |  |
| **Country** |  |  |
| **Ethnicity** |  |  |
| **Age (mean/median & range)** |  |  |
| **Sex/Gender** |  |  |
| **Sample size** |  |  |
| **Cancer diagnosis** |  |  |
| **Cancer survivorship definition** |  |  |
| **Education** |  |  |
| **Income** |  |  |
| **Data source/**  **Recruitment location**  **and methods** |  |  |

| **Measurement/ Exposure:** | | | |
| --- | --- | --- | --- |
|  | **Measurement** | **What has been measured** | **Data collection** / **Timing of measurement** |
| **Measurement of CVH / CVD risks** |  |  |  |
| **Other determinants** |  |  |  |
| **Main findings:** | | | |
| **Outcomes** | **Related to CVH (+/-/Not related)** | **Key findings and magnitude** | **Note** |
|  |  |  |  |
